# Mildly Elevated Serum Bilirubin Levels Are Negatively Associated with Carotid Atherosclerosis among Elderly Persons

**DOI:** 10.1371/journal.pone.0114281

**Published:** 2014-12-05

**Authors:** Ryuichi Kawamoto, Daisuke Ninomiya, Yoichi Hasegawa, Yoshihisa Kasai, Tomo Kusunoki, Nobuyuki Ohtsuka, Teru Kumagi, Masanori Abe

**Affiliations:** 1 Department of Community Medicine, Ehime University Graduate School of Medicine, Ehime, Japan; 2 Department of Internal Medicine, Seiyo Municipal Nomura Hospital, Ehime, Japan; Faculty of Biochemistry, Poland

## Abstract

Serum bilirubin may have a beneficial role in preventing oxidative changes in atherosclerosis. Limited information is available on whether serum total bilirubin is an independent confounding factor for carotid atherosclerosis {for example, intima-media thickness (IMT), plaque} measured noninvasively by B-mode ultrasonography only among elderly persons. The study subjects were 325 men aged 79±8 (mean ± standard deviation) years and 509 women aged 81±8 years that were enrolled consecutively from patients aged ≥60 years in the medical department. Carotid IMT and plaque were derived via B-mode ultrasonography. Multiple linear regression analysis showed that in men age (*β* = 0.199, *p* = 0.002), smoking status (*β* = 0.154, *p* = 0.006), GGT (*β* = -0.139, *p* = 0.039), and GGT (*β* = -0.133, *p* = 0.022) were significantly and independently associated with carotid IMT, and in women age (*β* = 0.186, *p*<0.001), systolic blood pressure (*β* = 0.104, *p* = 0.046), diastolic blood pressure (*β* = -0.148, *p* = 0.004), prevalence of antihypertensive medication (*β* = 0.126, *p* = 0.004), fasting plasma glucose (*β* = 0.135, *p* = 0.003), GGT (*β* = -0.104, *p* = 0.032), estimated glomerular filtration rate, serum bilirubin (*β* = -0.119, *p* = 0.006), and prevalence of cardiovascular disease (CVD) (*β* = 0.103, *p* = 0.017) were also independently associated with carotid IMT. The odds ratios (ORs) {95% confidence interval (CI)} of increasing serum bilirubin category were negatively associated with carotid IMT ≥1.0 mm and plaque in both genders. Compared to subjects with a serum bilirubin of Quartile-1, the multivariate-OR (95% CI) of carotid plaque was 0.25 (0.11–0.57) in the Quartile-4 male group, and 0.41 (0.21–0.78) in the Quartile-2 female group, 0.51 (0.26–0.98) in the Quartile-3 female group, and 0.46 (0.24–0.89) in the Quartile-4 female group. Our data demonstrated an independently negative association between serum bilirubin and carotid atherosclerosis in both genders.

## Introduction

Serum bilirubin, a major intravascular product of heme catabolism, is an endogenous compound that can be toxic in infants under certain conditions like excessive production of bilirubin due to hemolysis [1], but in adults a potent physiological antioxidant compound that may provide important protection against cardiovascular disease (CVD) and inflammation [2], [3]. It is generally suggested that oxidative reactions are involved in the pathophysiology of CVD processes [2], [4], [5], and that mildly increased bilirubin may have a physiologic function to protect against disease processes that involve oxygen and peroxyl radicals [6], [7].

The first report of a negative relationship between serum bilirubin levels and coronary artery disease was published as early as 1994 [8]. Since then, some studies have demonstrated that subjects with lower bilirubin levels have an increased risk of coronary artery calcification [9], ischemic stroke [10]. In a recent Taiwanese prospective study on patients with cardiac syndrome X followed for 5 years, in which patients with the lowest serum bilirubin levels had a higher incidence of non-fatal myocardial infarction, ischemic stroke, rehospitalization for unstable angina, and coronary revascularization procedures [11]. The same association was also reported in a recent United Kingdom prospective study, in which patients with the lower serum bilirubin levels had a higher incidence of CVD and death in both genders [12]. Meta analysis of studies focused on the association between serum bilirubin and atherosclerosis, an increase in serum bilirubin was associated with a decrease in CVD risk [3]. Although subjects in these studies include young persons, to our knowledge, there are few studies of which subjects are only elderly persons.

Carotid atherosclerosis {e.g., intima-media thickness (IMT), plaque} is an important and sensitive surrogate marker of CVD and can now be measured noninvasively by B-mode ultrasonography [13]–[15]. We have shown that this parameter is strongly associated with conventional cardiovascular risk factors, including age, central obesity, smoking status, metabolic syndrome (MetS), hypertension, hypertriglycerides, low high-density lipoprotein cholesterol (HDL-C) level, increased low-density lipoprotein cholesterol (LDL-C) level, uric acid, and diabetes [16]-[18]. However, limited information is available on whether serum bilirubin is an independent confounding factor for carotid atherosclerosis only among elderly persons by gender [19], [20].

Firstly, this study investigated serum bilirubin levels and their relation to potential confounding factors such as hypertension, hyperglycemia and lipids. Secondly, this study investigated whether there is an independent association of serum bilirubin with a direct and early measure of carotid atherosclerosis by B-mode ultrasonography. To examine these two issues, cross-sectional data from elderly persons were used.

## Materials and Methods

### Subjects

Subjects for this investigation were recruited from among consecutive elderly patients aged ≥60 years that visited the internal medical department of Seiyo Municipal Nomura Hospital. Participants with serum total bilirubin ≥2.1 mg/dL or alanine transaminase (ALT) ≥80 IU/L or gamma glutamyl transpeptidase (GGT) ≥80 IU/L were excluded to avoid confounding factors due to the high possibility of potential Gilbert syndrome and hepatobiliary disease. We additionally excluded participants with severe cardio-renal failure or nutritional disorders that would affect blood pressure, lipid and glucose metabolisms were also excluded. Exclusion criteria were severe hypotension as defined by systolic blood pressure (SBP) <80 mmHg; renal failure with an estimated glomerular filtration rate (eGFR) <30 mL/min/1.73 m^2^; total cholesterol (T-C) <100 mg/dL; and fasting plasma glucose (FPG) of <55 mg/dL or ≥400 mg/dL. Thus, 834 (men, 325/women, 509) patients were enrolled in the study. The main causes of consultations of the patients were respiratory disorders including pneumonia in 98/107 (men/women) cases, metabolic and endocrine diseases in 51/72, stroke in 49/55, cardiovascular disease other than stroke in 29/65, digestive disorders in 35/37, neurological disorders other than stroke in 18/61, infection in 7/27, urinary or reproductive organ disorders in 3/26, neoplasm in 16/12, collagen or skin disorders in 5/10, mental disorders in 3/6, hematological disorders in 3/2, miscellaneous diseases in 8/29. All procedures were approved by the Ethics Committee of Seiyo Municipal Nomura Hospital, and written informed consent was obtained from each participants.

### Evaluation of Confounding Factors

Information on demographic characteristics and confounding factors was collected using the medical records in all cases. Body mass index (BMI) was calculated by dividing weight (in kilograms) by the square of the height (in meters). Blood pressure was measured one time in the seated position by a nurse using a standard mercury sphygmomanometer or automatic manometer. Smoking status was defined as the number of cigarette packs per day multiplied by the number of years smoked (pack·year) irrespective of the difference between current and past smoking status: never, light (<20 pack ▪ year), moderate (20–39 pack ▪ year), and heavy (≥40 pack ▪ year). Blood samples were drawn after a 10 hour fast in the morning. T-C, triglycerides (TG), high-density lipoprotein cholesterol (HDL-C), FPG, creatinine (enzymatic method), ALT, GGT, and serum total bilirubin were measured within 24 hours after admission. Low-density lipoprotein cholesterol (LDL-C) level was calculated by the Friedewald formula [21]. Patients with TG levels of ≥400 mg/dL were excluded. Glomerular filtration rate was estimated by the following equation: Egfr  = 0.741×175×Cr^−1.154^× Age^−0.203^×0.742 (if female) [22], [23]. Histories of antihypertensive, antidyslipidemic, and antidiabetic medication use were also evaluated. Moreover, ischemic stroke, ischemic heart disease, and peripheral vascular disease were defined as CVD.

### Ultrasound image analysis

An ultrasonograph (Hitachi EUB-565, Aloka SSD-2000, or Prosound-α6 ) equipped with a 7.5 MHz linear type B-mode probe was used by a specialist in ultrasonography to evaluate sclerotic lesions of the common carotid arteries on a day close to the day of blood biochemistry analysis (within 2 days). Patients were asked to assume a supine position, and the bilateral carotid arteries were observed obliquely from the anterior and posterior directions. We measured the thickness of the intima-media complex (IMT) on the far wall of the bilateral common carotid artery about 10 mm proximal to the bifurcation of the carotid artery (as the image at that site is more clearly depicted than that near the wall) [24], [25] as well as the wall thickness near the 10 mm point on a B-mode monitor (Figure S1). We then used the mean value for analysis. Carotid plaques were considered as localized thickening and the echo luminance included those equal to high echogenic structures encroaching into the vessel lumen through the common carotid arteries to the carotid bifurcation [24]. Carotid atherosclerosis was defined as IMT ≥1.0 mm or plaque lesion [24], [25], [26].

### Statistical analysis

All values are expressed as the mean ± standard deviation (SD), unless otherwise specified, and in the cases of parameters with non-normal distribution (such as TG, FPG, ALT, GGT, and serum bilirubin), the data are shown as median (interquartile range) values. In all the analyses, parameters with non-normal distributions were used after log-transformation. Statistical analysis was performed using IBM SPSS Statistics Version 21 (Statistical Package for Social Science Japan, Inc., Tokyo, Japan). The differences in means and prevalence among the groups were analyzed by Student's t-test for continuous data and χ^2^ test for categorical data, respectively. Pearson's correlations were calculated in order to characterize the associations between various characteristics and carotid IMT. Forward stepwise multiple linear regression analysis (*p*-value for entry was <0.05 and for exit was >0.10) was used to evaluate the contribution of each confounding factor to carotid IMT. Subjects were divided into four groups based on sex-specific quartiles of serum bilirubin (men, Quartile-1, 0.13–0.54; Quartile-2, 0.55–0.73; Quartile-3, 0.74–1.02; Quartile-4, 1.03–2.00 mg/dL and women, Quartile-1, 0.21–0.51; Quartile-2, 0.52–0.67; Quartile-3, 0.68–0.88; Quartile-4, 0.89–2.00 mg/dL), and logistic regression analyses were used to test significant determinants of carotid IMT or plaque serving as the dichotomous outcome variable. A value of p<0.05 was considered significant.

## Results

### Characteristics of subjects by gender

Table 1 shows the background characteristics by gender. Several characteristics differed between men and women. Smoking status, prevalence of antidiabetic medication, eGFR, ALT, GGT, serum bilirubin, prevalence of ischemic stroke, and carotid IMT were higher in men than in women. Age, SBP, prevalence of antihypertensive medication, TG, HDL-C, LDL-C, and prevalence of antidyslipidemic medication were higher in women than in men. There were no inter-group differences in BMI, DBP, FPG, prevalence of ischemic heart disease, and prevalence of plaque.

**Table 1:** Characteristics of subjects by gender.

Characteristics	Men N = 325	Women N = 509	*P*-value*
Age (years)	79±8	81±8	**0.006**
Body mass index† (kg/m^2^)	21.2±3.5	21.5±3.8	0.280
Smoking status‡, N	148/14/23/140	502/1/1/5	**<0.001**
Systolic blood pressure (mmHg)	135±26	140±23	**0.004**
Diastolic blood pressure (mmHg)	75±14	77±14	0.280
Antihypertensive medication, N (%)	152 (46.8)	300 (58.9)	**0.001**
Triglycerides (mg/dL)	73 (59–97)	79 (62–109)	**0.036**
HDL cholesterol (mg/dL)	54±18	57±16	**0.003**
LDL cholesterol (mg/dL)	101±34	111±35	**<0.001**
Antidyslipidemic medication, N (%)	16 (4.9)	52 (10.2)	**0.006**
Fasting plasma glucose (mg/dL)	119 (100–146)	114 (99–142)	0.332
Antidiabetic medication, N (%)	69 (21.2)	76 (14.9)	**0.024**
eGFR § (mL/min/1.73 m^2^)	66.3±21.5	60.8±19.1	**<0.001**
Alanine aminotransferase (IU/L)	16 (11–22)	14 (10–19)	**0.006**
Gamma-glutamyltransferase (IU/L)	23 (16–34)	16 (12–24)	**<0.001**
Serum bilirubin (mg/dL)	0.73 (0.54–1.03)	0.68 (0.52–0.89)	**0.023**
Cardiovascular disease, N (%)	151 (46.5)	210 (41.3)	0.152
Ischemic stroke, N (%)	136 (41.8)	177 (34.8)	**0.040**
Ischemic heart disease, N (%)	30 (9.2)	52 (10.2)	0.721
Carotid intima-media thickness (mm)	1.05±0.22	0.98±0.21	**<0.001**
Plaque, N (%)	243 (74.8)	356 (69.9)	0.135

Data are means ± standard deviation. HDL, high-density lipoprotein; LDL, low-density lipoprotein; eGFR, estimated glomerular filtration rate. †Body mass index was calculated using weight in kilograms divided by the square of the height in meters. ‡Smoking status: daily consumption (pack) × duration of smoking (year) {never, light (<20 pack ▪ year), moderate (20–39 pack ▪ year), heavy (≥40 pack ▪ year)}. §eGFR was estimated by the following equation:  = 0.741×175×Cr^−1.154^× Age^−0.203^×0.742 (if female). Data for triglycerides, fasting plasma glucose, alanine aminotransferase, gamma-glutamyltransferase, and serum bilirubin were skewed and are presented as median (interquartile range) values, and were log-transformed for analysis. *Student's t-test was used for the continuous data and χ^2^ test for the categorical data.

### Relationship between serum bilirubin and variables within each gender

Table 2 shows the relationship between serum bilirubin and various confounding factors within each gender. In men, serum bilirubin correlated positively with DBP and HDL-C. In women, serum bilirubin correlated positively with BMI, while negatively with TG.

**Table 2:** Relationship between serum bilirubin and variables within each gender.

	Men N = 325	Women N = 509
Characteristics	r (*P*-value)	r (*P*-value)
Age	−0.061 (0.271)	−0.073 (0.100)
Body mass index	0.096 (0.084)	**0.087 (0.049)**
Smoking status	0.087 (0.118)	0.022 (0.625)
Systolic blood pressure	0.064 (0.249)	−0.006 (0.884)
Diastolic blood pressure	**0.143 (0.010)**	0.041 (0.357)
Antihypertensive medication (No = 0, Yes = 1)	−0.048 (0.391)	0.028 (0.526)
Triglycerides	−0.078 (0.158)	−**0.181 (<0.001)**
HDL cholesterol	**0.191 (0.001)**	0.079 (0.076)
LDL cholesterol	0.068 (0.222)	0.068 (0.124)
Antidyslipidemic medication (No = 0, Yes = 1)	−0.024 (0.669)	0.036 (0.413)
Fasting plasma glucose	0.085 (0.128)	−0.037 (0.400)
Antidiabetic medication (No = 0, Yes = 1)	0.027 (0.626)	−0.010 (0.814)
eGFR	0.107 (0.053)	0.035 (0.427)
Serum uric acid	−0.078 (0.162)	−0.030 (0.500)
Alanine aminotransferase	−0.001 (0.986)	0.012 (0.789)
Gamma-glutamyltransferase	0.045 (0.421)	0.067 (0.130)
Cardiovascular disease	−0.070 (0.209)	−0.030 (0.493)

r, Pearson**'**s correlation coefficient. Data for triglycerides, fasting plasma glucose, alanine aminotransferase, gamma-glutamyltransferase, and serum bilirubin were skewed and log-transformed for analysis.

### Relationship between variables including serum bilirubin and carotid intima-media thickness within each gender

Table 3 shows the relationship between the background characteristics and carotid IMT within each gender. In men, age, smoking status, prevalence of antihypertensive medication, and prevalence of CVD correlated positively with carotid IMT, while GGT correlated negatively with carotid IMT. In women, age, prevalence of antihypertensive medication, FPG, and prevalence of CVD correlated positively with carotid IMT, while DBP, GGT, serum bilirubin correlated negatively with carotid IMT.

**Table 3:** Relationship between variables including serum bilirubin and carotid intima-media thickness within each gender.

	Men N = 325	Women N = 509
Characteristics	r (*P*-value)	r (*P*-value)
Age	**0.261 (<0.001)**	**0.224 (<0.001)**
Body mass index	−0.088 (0.115)	0.030 (0.507)
Smoking status	**0.112 (0.043)**	0.039 (0.386)
Systolic blood pressure	0.085 (0.128)	0.046 (0.297)
Diastolic blood pressure	0.016 (0.769)	−**0.098 (0.027)**
Antihypertensive medication (No = 0, Yes = 1)	**0.140 (0.012)**	**0.123 (0.006)**
Triglycerides	−0.034 (0.542)	−0.002 (0.964)
HDL cholesterol	−0.101 (0.069)	−0.060 (0.178)
LDL cholesterol	−0.004 (0.942)	0.027 (0.545)
Antidyslipidemic medication (No = 0, Yes = 1)	0.003 (0.956)	−0.080 (0.072)
Fasting plasma glucose	−0.021 (0.704)	**0.126 (0.004)**
Antidiabetic medication (No = 0, Yes = 1)	−0.018 (0.747)	0.021 (0.641)
eGFR	−0.086 (0.123)	−0.015 (0.737)
Alanine aminotransferase	−0.095 (0.086)	−0.049 (0.269)
Gamma-glutamyltransferase	−**0.182 (0.001)**	−**0.140 (0.002)**
Serum bilirubin	−0.068 (0.224)	−**0.132 (0.003)**
Cardiovascular disease	**0.158 (0.004)**	**0.144 (0.001)**

r, Pearson**'**s correlation coefficient. Data for triglycerides, fasting plasma glucose, alanine aminotransferase, gamma-glutamyltransferase, and serum bilirubin were skewed and log-transformed for analysis

**Figure 1:**
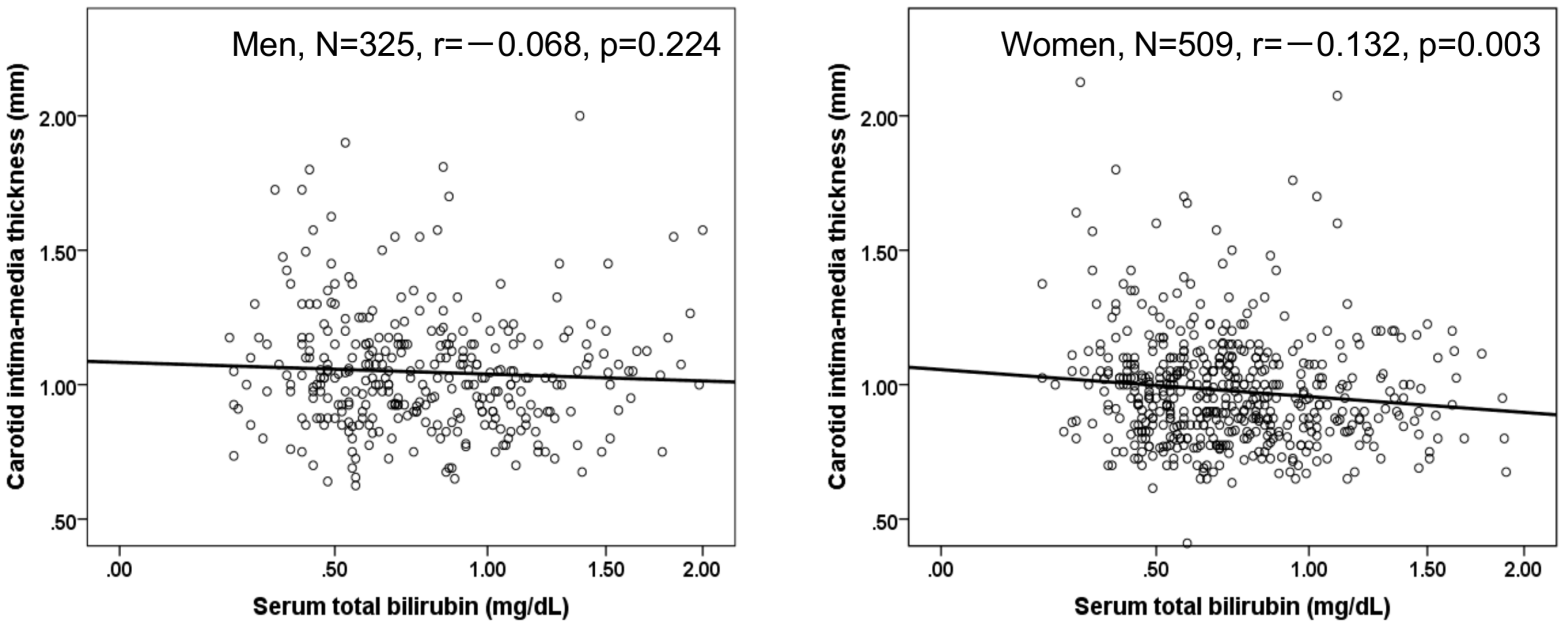
Relationship between serum total bilirubin and carotid intima-media thickness within each gender.

Figure 1 shows the correlation between serum bilirubin and carotid IMT within gender. The correlation coefficient between the serum bilirubin and carotid IMT was significant only in women.

### Multiple linear regression analysis of variables including serum bilirubin for carotid intima-media thickness within each gender

To find independently confounding factors for carotid IMT, in Table 4 multiple linear regression analysis showed that in men age and smoking status, GGT (*β* = -0.139), and prevalence of CVD (*β* = 0.133) were significantly and independently associated with carotid IMT, and in women age (*β* = 0.186), SBP (*β* = 0.104), DBP (*β* = -0.148), prevalence of antihypertensive medication (*β* = 0.126), FPG (*β* = 0.135), GGT (*β* = -0.104), serum bilirubin (*β* = -0.119), and prevalence of CVD (*β* = 0.103) were also independently associated with carotid IMT. In male model 2, age (*β* = 0.232) and smoking status (*β* = 0.155), SBP (*β* = 0.106), GGT (*β* = -0.136), and prevalence of CVD (*β* = 0.131) was significantly and independently associated with carotid IMT, while in female model 2, serum bilirubin (*β* = -0.107) as well as age (*β* = 0.168), prevalence of antihypertensive medication (*β* = 0.115), FPG (*β* = 0.134), GGT (*β* = -0.122), and prevalence of CVD (*β* = 0.093) was significantly and independently associated with carotid IMT.

**Table 4:** Multiple linear regression analysis of variables including serum bilirubin for carotid intima-media thickness within each gender.

	Men N = 325		Women N = 509	
	Multiple linear regression analysis		Multiple linear regression analysis	
	Model 1	Model 2	Model 1	Model 2
Characteristics	β(*P*-value)	β(*P*-value)	β(*P*-value)	β(*P*-value)
Age	**0.199 (0.002)**	**0.232 (<0.001)**	**0.186 (<0.001)**	**0.168 (<0.001)**
Body mass index	−0.064 (0.312)	–	0.071 (0.123)	–
Smoking status	**0.154 (0.006)**	**0.155 (0.004)**	0.069 (0.101)	–
Systolic blood pressure	0.072 (0.331)	**0.106 (0.047)**	**0.104 (0.046)**	–
Diastolic blood pressure	0.059 (0.420)	–	**−0.148 (0.004)**	–
Antihypertensive medication (No = 0, Yes = 1)	0.087 (0.125)	–	**0.126 (0.004)**	**0.115 (0.007)**
Triglycerides	−0.008 (0.906)	–	−0.050 (0.317)	–
HDL cholesterol	−0.084 (0.144)	–	−0.044 (0.331)	–
LDL cholesterol	0.029 (0.619)	–	0.081 (0.086)	–
Antidyslipidemic medication (No = 0, Yes = 1)	−0.027 (0.627)	–	−0.083 (0.054)	–
Fasting plasma glucose	0.039 (0.519)	–	**0.135 (0.003)**	**0.134 (0.002)**
Antidiabetic medication (No = 0, Yes = 1)	0.021 (0.723)	–	−0.031 (0.497)	–
eGFR	−0.057 (0.324)	–	0.074 (0.104)	–
Alanine aminotransferase	−0.019 (0.750)	–	0.007 (0.877)	–
Gamma-glutamyltransferase	**−0.139 (0.039)**	**−0.136 (0.016)**	**−0.104 (0.032)**	**−0.122 (0.005)**
Serum bilirubin	-0.041 (0.461)	**–**	**−0.119 (0.006)**	**−0.107 (0.011)**
Cardiovascular disease	**0.133 (0.022)**	**0.131 (0.014)**	**0.103 (0.017)**	**0.093 (0.031)**
R^2^	**0.157 (<0.001)**	**0.132 (<0.001)**	**0.153 (<0.001)**	**0.113 (<0.001)**

β, standardized coefficient; R^2^, multiple coefficient of determination. Model 1, forced entry method and Model 2, forward stepwise method. Data for triglycerides, fasting plasma glucose, alanine aminotransferase, gamma-glutamyltransferase, and serum bilirubin were skewed and log-transformed for analysis.

### Odds ratios for carotid atherosclerosis by serum bilirubin categories within each gender

Table 5 shows the odds ratios (ORs) {95% confidence interval (CI)} of carotid IMT ≥1.0 mm including plaque and carotid plaque with increasing serum bilirubin categories. Increasing serum total bilirubin categories were negatively associated with carotid atherosclerosis in both genders. Compared to subjects with a serum bilirubin of Quartile-1 (men, 0.13–0.54 mg/dL and women, 0.21–0.51 mg/dL), the multivariate-OR (95% CI) of carotid plaque was 0.25 (0.11–0.57) in the Quartile-4 male group (1.03–2.00 mg/dL), and 0.41 (0.21–0.78) in the Quartile-2 female group (0.52–0.67 mg/dL), 0.51 (0.26–0.98) in the Quartile-3 female group (0.68–0.88 mg/dL), and 0.46 (0.24–0.89) in the Quartile-4 female group (0.86–2.00 mg/dL).

**Table 5:** Odds ratios for carotid atherosclerosis by serum bilirubin quartiles within each gender.

	Men N = 325					Women N = 509				
Characteristics	N	Non-adjusted odds ratio (95%CI)	*P-value*	Multivariate-adjustedodds ratio (95%CI)§	*P-value*	N	Non-adjusted odds ratio (95%CI)	*P-value*	Multivariate-adjusted odds ratio (95%CI)§	*P-value*
Carotid IMT ≥1.0 mm including plaque										
Quartile-1	88	1.00		1.00		121	**1.00**		1.00	
Quartile-2	75	0.40 (0.16–1.01)	0.051	0.38 (0.14–1.06)	0.065	128	**0.35 (0.18**–**0.65)**	**0.001**	**0.40 (0.20**–**0.83)**	**0.013**
Quartile-3	79	0.43 (0.17–1.07)	0.069	0.43 (0.15–1.20)	0.107	130	0.65 (0.34–1.28)	0.213	0.61 (0.29–1.30)	0.201
Quartile-4	83	**0.28 (0.12**–**0.67)**	**0.004**	**0.29 (0.11**–**0.77)**	**0.013**	130	**0.38 (0.20**–**0.72)**	**0.003**	**0.43 (0.21**–**0.89)**	**0.022**
Carotid plaque										
Quartile-1	88	1.00		1.00		121	1.00		1.00	
Quartile-2	75	0.55 (0.25–1.21)	0.138	0.47 (0.19–1.15)	0.097	128	**0.37 (0.21**–**0.66)**	**0.001**	**0.41 (0.21**–**0.78)**	**0.007**
Quartile-3	79	0.59 (0.27–1.29)	0.186	0.53 (0.21–1.31)	0.169	130	0.56 (0.31–1.02)	0.057	0.51 (0.26–0.98)	**0.045**
Quartile-4	**83**	**0.26 (0.13**–**0.55)**	**<0.001**	**0.25 (0.11**–**0.57)**	**0.001**	130	**0.41 (0.23**–**0.73)**	**0.003**	**0.46 (0.24**–**0.89)**	**0.021**

CI, confidence interval. § Adjusted for confounding factors in model 1 of Table 4 by stepwise multiple logistic regression analysis. Subjects were divided into four groups based on quartile of serum bilirubin (men, Quartile-1, 0.13–0.54; Quartile-2, 0.55–0.73; Quartile-3, 0.74–1.02; Quartile-4, 1.03–2.00 mg/dL and women, Quartile-1, 0.21–0.51; Quartile-2, 0.52–0.67; Quartile-3, 0.68–0.88; Quartile-4, 0.89–2.00 mg/dL).

## Discussion

To examine any possible contribution of serum bilirubin to carotid atherosclerosis, we studied the relationship between CVD confounding factors, including serum bilirubin, and carotid IMT and plaque. This study showed that serum bilirubin was independently and negatively related to carotid atherosclerosis evaluated by ultrasonography. Increased carotid IMT occurred in parallel with the decline in serum bilirubin in women, and serum bilirubin quartiles are significantly and negatively associated with carotid IMT ≥1.0 mm including plaque and plaque, independent of other confounding factors in both genders. To our knowledge, this is the first study to indicate a negative relationship between serum bilirubin and carotid atherosclerosis among Japanese elderly persons.

There are few reports which studied the association between serum bilirubin and carotid atherosclerosis and they are all based on cross-sectional analysis. In healthy 111 men without manifested atherosclerosis, the mean IMT and age-adjusted odds ratio for carotid atherosclerosis (IMT >50% of controls) in hyperbilirubinemic subjects with Gilbert syndrome were significantly lower than that of controls [20]. Among 1,741 subjects enrolled in the study, Ishizaki et al. found that 330 subjects (19%) were found to have carotid plaque in either or both of the carotid arteries, and an odds ratio of 0.37 for carotid plaque was associated with an increase of 1.0 mg/dL in serum bilirubin concentration [27]. From 91 healthy participants aged 25-45 years, Erdogan et al. demonstrated that carotid IMT was significantly greater in both genders with lower serum bilirubin levels compared to those with elevated serum bilirubin levels (0.51**±**0.08 mm versus 0.41**±**0.08 mm, p<0.001; 0.55**±**0.12 mm versus 0.40**±**0.07 mm, *P* = 0.002, in women and men, respectively) [19]. In198 hypertensive patients (104 men) aged 46–82 years, serum bilirubin levels were independently associated with carotid atherosclerosis in women and men, with odds ratios of 0.49 (95% CI, 0.28-0.71) and 0.66 (95% CI, 0.46–0.80) [28]. Among 346 type 2 diabetic patients (146 men) aged 35–95 years, serum bilirubin levels were independently associated with carotid IMT (*β* = -0.34) [29]. In a 5-year prospectively study of 245 healthy individuals (98 men) aged 30–89 years at baseline, in subjects with plaque at entry, serum bilirubin as well as age, changes in eGFR, and the baseline levels of serum albumin dependently influenced the outcome of carotid plaque [30]. In 142 patients with coronary artery ectasia and newly diagnosed coronary artery disease, lower serum bilirubin had greater carotid IMT compared to controls with normal coronary angiograms [31]. In 417 patients without coronary artery disease, serum bilirubin levels were independently associated with aortic IMT (β = -0.513, *P*<0.001) [32]. Among 897 participants aged of ≥60 years, our study support the possibility that mildly elevated serum bilirubin levels could be implicated in the inhibition of atherosclerosis, especially in participants without CVD. Serum bilirubin may play an important role for early morbidity. Kao et al. [33] demonstrate a negative correlation between high serum bilirubin and increased functional dependence among elderly persons.

The precise mechanisms that lead to a reduction in carotid atherosclerosis in individuals with mildly elevated serum bilirubin levels are not completely understood. Several studies have demonstrated a negative relationship between serum bilirubin and the presence of CVD. In addition, serum bilirubin correlated inversely with several known risk factors for CVD, such as smoking, LDL-C, diabetes, and obesity, and correlated directly with HDL-C [7], [34], [35]. In our study, serum bilirubin in men correlated positively with BMI, DBP, and HDL-C, while negatively with TG. Serum bilirubin is a potent endogenous antioxidant that inhibits LDL oxidation, and bilirubin may limit lipid peroxidation and retard the progression of atherosclerosis [36], [37], [38]. Serum bilirubin exerts potential antioxidant and anti-inflammatory effects on the vasculature, acting against plaque formation and subsequent atherosclerosis [6], [39], [40], [41]. These properties appear to allow bilirubin to inhibit multiple steps in atherogenesis.

However, interpreting our results has several limitations that must be considered. Firstly, based on its cross-sectional study design, the present findings are inherently limited in the ability to eliminate causal relationships between confounding factors and carotid atherosclerosis. Secondly, since all subjects were patients, we could not eliminate the possible effects of underlying diseases (e.g., liver disease) and medications used for hypertension and dyslipidemia on the present findings. In our study, participants with serum total bilirubin ≥2.1 mg/dL or ALT ≥80 IU/L or GGT ≥80 IU/L were excluded, but individuals with Gilbert's syndrome and may be included in the Quartile-4 (men, 1.03–2.00 mg/dL and women, 0.89–2.00 mg/dL) group. Thirdly, in this study our model could not account only for 11.3–15.7% of the variance in the IMT results. Secondary prevention interventions in obesity, hypertension, dyslipidemia and diabetes mellitus may be successful in reducing confounding factors, thus attenuating the observed association of confounding factors with disease. Fourth, it is generally known that elderly persons normally eat less (especially if hospitalized) which leads inevitably to increased serum bilirubin levels [42]. In our study, serum bilirubin did not correlate with age. Therefore the demographics and referral source may limit generalizability.

## Conclusions

The present study showed that a lower serum bilirubin is strongly associated with an increased prevalence of carotid atherosclerosis in both genders. The underlying mechanism behind this relationship is unclear, but it seems to be independent of traditional cardiovascular confounding factors such as age, BMI, smoking status, hypertension, dyslipidemia, uric acid, diabetes and liver enzymes. Serum bilirubin in addition to the consideration of present conventional confounding factors may be useful for individual risk assessment. Further prospective population-based studies are needed to investigate the mechanisms underlying this association.

## Supporting Information

Figure S1 **Ultrasound images of common carotid artery.**
(XLSX)

Data S1 **Data of subjects.**
(PDF)
