# Supplementary figures and images for: Mildly Elevated Serum Bilirubin Levels Are Negatively Associated with Carotid Atherosclerosis among Elderly Persons
Source: PLoS One. 2014 Dec 5;9(12):e114281. doi: 10.1371/journal.pone.0114281 (PMC4257609; doi:10.1371/journal.pone.0114281)

# Carotid intima-media thickness

Salonen 1

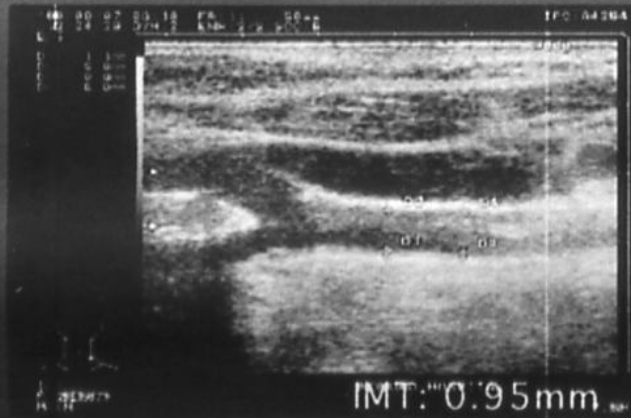

Salonen 2

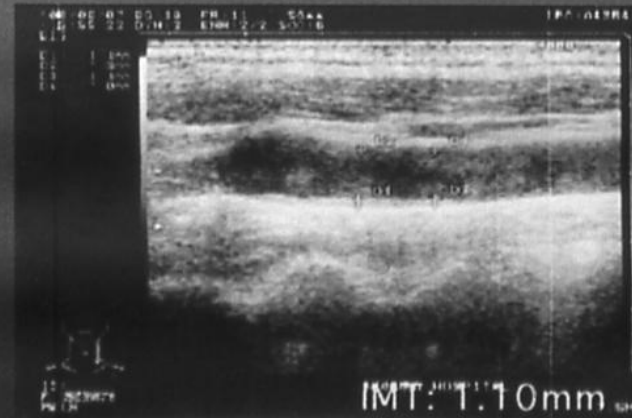

Salonen 3

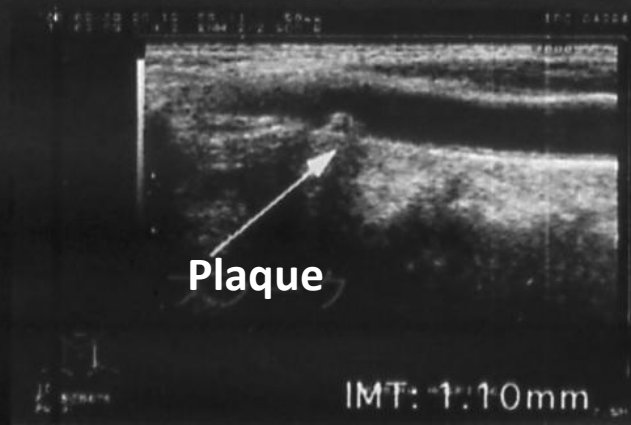

Salonen 4

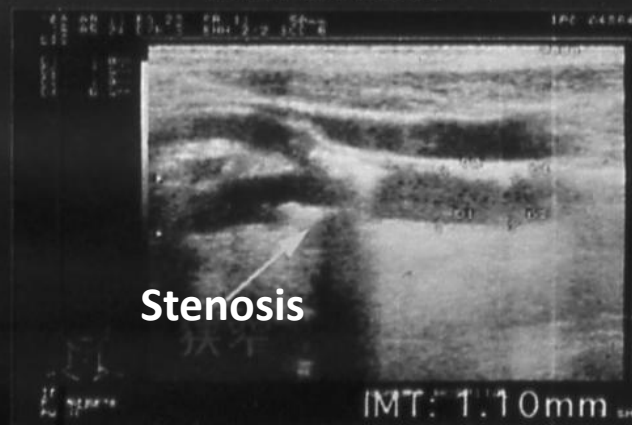

Supplement: Figure S1 — Ultrasound images of common carotid artery. (XLSX) [file pone.0114281.s001.xlsx]
